# Co-operation of TLR4 and raft proteins in LPS-induced pro-inflammatory signaling

**DOI:** 10.1007/s00018-014-1762-5

**Published:** 2014-10-22

**Authors:** Agnieszka Płóciennikowska, Aneta Hromada-Judycka, Kinga Borzęcka, Katarzyna Kwiatkowska

**Affiliations:** Laboratory of Molecular Membrane Biology, Nencki Institute of Experimental Biology, 3 Pasteur St., 02-093 Warsaw, Poland

**Keywords:** Lipopolysaccharide, Toll-like receptor 4, Plasma membrane rafts, CD14, Lyn tyrosine kinase, Acid sphingomyelinase, CD36

## Abstract

Toll-like receptor 4 (TLR4) is activated by lipopolysaccharide (LPS), a component of Gram-negative bacteria to induce production of pro-inflammatory mediators aiming at eradication of the bacteria. Dysregulation of the host responses to LPS can lead to a systemic inflammatory condition named sepsis. In a typical scenario, activation of TLR4 is preceded by binding of LPS to CD14 protein anchored in cholesterol- and sphingolipid-rich microdomains of the plasma membrane called rafts. CD14 then transfers the LPS to the TLR4/MD-2 complex which dimerizes and triggers MyD88- and TRIF-dependent production of pro-inflammatory cytokines and type I interferons. The TRIF-dependent signaling is linked with endocytosis of the activated TLR4, which is controlled by CD14. In addition to CD14, other raft proteins like Lyn tyrosine kinase of the Src family, acid sphingomyelinase, CD44, Hsp70, and CD36 participate in the TLR4 signaling triggered by LPS and non-microbial endogenous ligands. In this review, we summarize the current state of the knowledge on the involvement of rafts in TLR4 signaling, with an emphasis on how the raft proteins regulate the TLR4 signaling pathways. CD14-bearing rafts, and possibly CD36-rich rafts, are believed to be preferred sites of the assembly of a multimolecular complex which mediates the endocytosis of activated TLR4.

## Introduction

Toll-like receptors (TLRs) recognize a variety of microbial structural components called pathogen-associated molecular patterns (PAMPs). Upon recognition of the PAMPs, TLRs trigger production of pro-inflammatory mediators helping to eradicate infection. Until now, thirteen TLRs have been identified and described in mammals, twelve of which are expressed in mice and ten in humans. The discovery of the role of TLRs has greatly advanced the field of innate immunology and was honored with the Nobel Prize to Jules Hoffmann and Bruce Beutler in 2011. The Beutler’s group has revealed that TLR4 is activated by lipopolysaccharide (LPS, endotoxin), a component of the outer membrane of Gram-negative bacteria. LPS is anchored in the bacterial membrane by up to seven acyl chains composing so-called lipid A which is bound to an oligosaccharide core and a highly variable polysaccharide chain named *O*-antigen. Lipid A is the most evolutionarily conserved part of LPS responsible for its pro-inflammatory activity. The maximal potency to trigger inflammation is shown by LPS with a bis-phosphorylated lipid A composed of six saturated acyl chains. The pro-inflammatory action of LPS is crucial for curbing bacterial infections, but excessive host responses to LPS can lead to systemic inflammatory conditions—sepsis, severe sepsis, and fatal septic shock. The incidence of severe sepsis in the European Union has been estimated at 90.4 cases per 100,000 population. In the United States, severe sepsis causes approximately 215,000 deaths per year (nearly as many as lung, colorectal, and breast cancers together). The mortality of severe sepsis reaches 30–50 % worldwide and the absence of efficient therapies makes studies on the molecular mechanisms of activation of cells by LPS of utmost importance. Furthermore, the pro-inflammatory activity of TLR4 is linked with pathological responses to endogenous ligands in autoimmune disorders and chronic inflammatory conditions accompanying development of atherosclerosis, neurodegenerative diseases, and others [1–3], which fuels interest in TLR4 signaling.

## Activation of TLR4 by LPS

Since the identification of TLR4 as the LPS receptor in 1998, it has long been assumed to trigger all the responses to LPS [4, 5]. The receptor is expressed in myeloid lineage cells and some non-immune cells, like intestinal epithelial cells and endothelial cells. It is a single-spanning transmembrane protein with an extracellular domain composed of 22 leucine-rich repeats conferring a horseshoe-like shape on the protein, found typical for TLRs by crystallography studies [6–8]. A transmembrane helix of 21 amino acids links the TLR4 ectodomain with the endodomain of about 200 amino acids, which contains a conserved region called the Toll/IL-1 receptor (TIR) domain. The TIR domain is critical for signal transduction and is also present in adaptor proteins of TLRs [9].

**Fig. 1:**
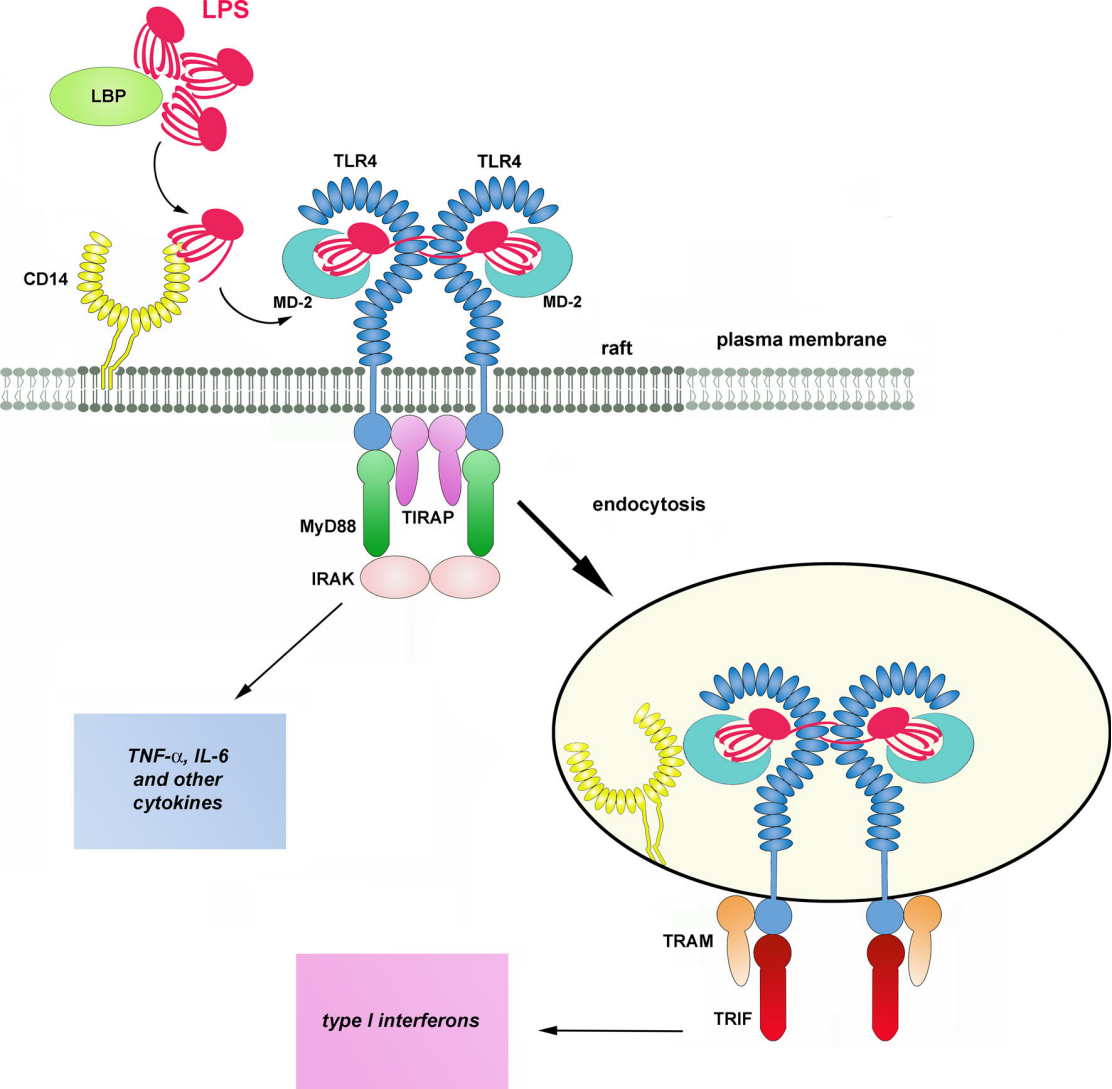
Activation of TLR4 by LPS. LBP facilitates transfer of LPS monomers to CD14 with the help of LBP and CD14 subsequently shifts the endotoxin to TLR4/MD-2 complex. Dimerization of the receptor complex induces the assembly of TIRAP, MyD88, and IRAK kinases in a myddosome at the TIR domain of TLR4 inducing a signaling pathway leading to production of pro-inflammatory cytokines. After endocytosis, TRAM and TRIF associate with TLR4 triggering a signaling pathway which controls production of type I interferons and some other cytokines. The presence of leucine-rich repeats in CD14 and TLR4 is marked by *ellipses*. The molecules are drawn not to scale

In a typical scenario, activation of TLR4 requires a cascade of events starting from an interaction of LPS with LPS-binding protein (LBP) in the serum (Fig. 1). LBP binds to LPS-rich membranes of bacteria and LPS aggregates (micelles) formed by this amphipatic molecule in aqueous solutions. LBP facilitates extraction of LPS monomers by CD14 protein most likely by changing the arrangement of LPS aggregates [10, 11]. CD14 is a GPI-anchored glycoprotein found on the surface of the plasma membrane of monocytes, macrophages, dendritic cells, and at a lower level, neutrophils [12, 13]. It is a horseshoe-shaped dimer containing a total of 22 leucine-rich repeats. The main site involved in the binding of LPS (and possibly of other acylated ligands) is located in a large N-terminal highly hydrophobic pocket of CD14 monomers [14, 15]. The transfer of LPS to CD14 is facilitated by albumin which shields the hydrophobic lipid A when LPS moves across an aqueous milieu [16]. Subsequently, CD14 transfers the LPS to MD-2 in the TLR4/MD-2 complex, again with the assistance of albumin [17–19]. One fatty acid residue of LPS is expected to bind outside the pocket and to facilitate the association of CD14 with MD-2 [15].

Similarly to CD14, a hydrophobic pocket of MD-2 accommodates most of the lipid portion of LPS, however, one of the six acyl chains of the endotoxin is left outside the pocket and interacts with the ectodomain of a neighboring TLR4 molecule. Additionally, the phosphate groups of lipid A interact with positively charged amino acids of TLR4. By simultaneously binding to MD-2 and to the adjacent TLR4 receptor, LPS facilitates formation of “M” shaped dimers of TLR4/MD-2 complexes [8, 20–22]. The TLR4 dimerization facilitates recruitment of two pairs of adaptor proteins, TIRAP/MyD88 and TRAM/TRIF, to the TIR domain of the receptor by homotypic TIR–TIR interactions. MyD88 then recruits the IRAK4 and IRAK2 (or IRAK1) kinases in a hierarchic manner and a so-called myddosome is assembled [23, 24]. This multimolecular complex triggers a signaling cascade leading to early-phase activation of NFκB and MAP kinases and controls the production of pro-inflammatory cytokines like tumor necrosis factor- α (TNF-α), interleukin-6 (IL-6), etc. On the other hand, TRIF initiates a signaling pathway which activates IRF3 transcription factor, leading to the expression of type I interferons (IFN) and IFN-inducible chemokines like IL-10 and RANTES; late-phase activation of NFκB and MAP kinases follows [25]. Recent discoveries underscore the dichotomy of the MyD88- and TRIF-dependent signaling pathways of TLR4 demonstrating that the TRAM/TRIF adaptors are recruited to TLR4 after the receptor is endocytosed [26–28].

Lipopolysaccharide has been shown to induce co-clustering of TLR4 with CD14 and also with heat shock proteins 70 and 90 (Hsp70, Hsp90), CD55, CD11b/CD18, chemokine receptor 4 (CXRC4), Fcγ receptors and scavenger receptors (SR) [29–32]. Some of these proteins can function as LPS-binding proteins; however, signaling properties of CXCR4 and class B scavenger receptors, including CD36, CLA-1/SRB-I, and CLA-2/SRB-II, have been reported [33–35]. A contribution of scavenger receptor A (SR-A) to maximal NFκB activation and subsequent TNF-α production in LPS-stimulated macrophages has been shown, although the receptor also co-operates with CD14 in the uptake of large amounts of LPS, leading to its detoxification [32, 36]. Recent studies have unveiled an alternative, TLR4-independent, activation of pro-inflammatory responses to LPS. When high concentrations of LPS persist in the body, LPS can be aberrantly found in the cytoplasm of macrophages where it binds to murine caspase-11 (human caspase-4 and -5) and activates a non-canonical inflammasome leading to the generation of pro-inflammatory IL-1β and IL-11, and to pyroptosis [37–39].

Due to the complexity of the “LPS-sensing apparatus” in immune cells our understanding of LPS-induced signaling is still incomplete and likely oversimplified. A line of data indicates that an important factor governing TLR4 activation by LPS is the association of TLR4 and its accessory proteins with microdomains of the plasma membrane named rafts. In this review, we summarize the knowledge on the involvement of raft proteins which include CD14 and Lyn kinase in LPS-induced activation of cells. As an increasing body of data suggests that other raft proteins, like CD44 and CD36, co-operate with TLR4 in the induction of the pro-inflammatory responses not only to LPS but also to endogenous ligands, we address these issues following the presentation of LPS-induced responses. Several other aspects of TLR functioning, in particular those of TLR4, have been discussed in previous excellent reviews [1, 3, 25, 40].

## Rafts as platforms of TLR4 activation

Plasma membrane rafts are envisioned as nanoscale assemblies of saturated sphingolipids, cholesterol, and selected proteins which separate laterally for subsecond lifetime in the glycerophospholipid-rich milieu of the membrane [41, 42]. This concept of a dynamic heterogeneous plasma membrane organization has largely been accepted after years of studies and disputes taking place since the principles of raft assembly had been proposed [43]. The assembly of rafts is driven by preferential interactions between cholesterol and long saturated acyl chains of sphingolipids, and also by the capacity of sphingolipids for intermolecular hydrogen bonding. The process is mimicked by liquid ordered (*L*_o_) and disordered (*L*_d_) phase separation of lipids in model membranes which depends solely on cholesterol-sphingolipid interactions [42, 44–46]. However, the composition of the plasma membrane is far more complicated and multifarious lipid–lipid, lipid–protein, and protein–protein interactions affect the formation of nanoscopic raft domains and facilitate their clustering into more stable functional platforms at physiological conditions [47–49]. The inherent propensity of the plasma membrane components to form nanoscopic rafts has been demonstrated in studies using vesicles/spheres of the plasma membrane obtained by osmotic swelling or chemically induced vesiculation of cells. In these plasma membrane fragments, separation of *L*_o_ and *L*_d_ phases accompanied by accumulation of selected membrane proteins in the ordered phase can be observed [47, 49–51].

Several mechanisms facilitate the association of proteins with sphingolipid/cholesterol assemblies. Due to the high content of saturated acyl chains of sphingolipids in such assemblies, the thickness of the lipid bilayer and lipid packing increase locally in comparison with the surrounding bilayer composed mainly of unsaturated glycerophospholipids. This, in turn, creates conditions at which insertion of proteins modified with saturated lipids into those domains rather than into the membrane bulk is energetically favorable. For these reasons, in the outer leaflet of rafts, proteins with glycosylphosphatidylinositol (GPI) anchor, like CD14, are accumulated [52]. The inner leaflet of rafts preferentially accommodates proteins modified by palmitoylation, which include tyrosine kinases of the Src family and Gα subunits of trimeric G proteins [53]. Reciprocally, palmitoylated proteins can facilitate the assembly of rafts [54]. There are also some transmembrane proteins, mostly palmitoylated ones, that are intrinsically targeted to rafts, as exemplified by CD44 and CD36 involved in TLR4 signaling (see below) and Cbp/PAG and NTAL multipurpose adaptor proteins [55–59]. In fact, the presence of palmitoylation, the length and the amino acid sequence of the protein transmembrane domain, and the oligomerization status of the protein are now recognized as essential factors controlling the partition of transmembrane proteins to rafts [60, 61]. It has also been proposed that cholesterol- and sphingolipid-rich shells adjacent to the transmembrane domains of proteins facilitate their association with rafts [62]. In contrast, a transmembrane domain coupled to an unsaturated phosphatidylethanolamine can exclude the protein from rafts [49]. A combination of lipid–lipid and lipid–protein interactions in living cells is likely to give rise to plasma membrane rafts of different protein composition [49].

Raft occurrence is manifested upon cell stimulation, when they merge into larger platforms and facilitate interactions of some receptors with their proximal signaling molecules. This mode of action is common to immunoreceptors, including T cell receptor (TCR), Fcγ receptor IIa (FcγRIIa), B-cell receptor (BCR), and Fcε receptor I (FcεRI), which trigger signaling cascades after phosphorylation by raft-anchored tyrosine kinases of the Src family [57, 63–65]. Raft-based platforms also function in cell polarization and membrane trafficking from the Golgi apparatus to the plasma membrane, and during endocytosis (see [66, 67] for review). All these events are relevant to LPS-induced activation of macrophages drawing attention to rafts as potential sites of LPS interaction with CD14 and TLR4.

The plasma membrane rafts share lipid composition with caveolae, flask-shaped invaginations of the plasma membrane stabilized by caveolin 1–3 proteins. Contribution of caveolae to macrophage functioning is unclear, although depletion of caveolin-1 inhibited phagocytosis of *Escherichia coli*, decreased amounts of CD14, CD36, and TLR4, and reduced cytokine production in macrophages and *cav1* ^−*/*−^ mice exposed to the bacteria [68].

Ample data indicate that TLR4 and accessory proteins can associate with plasma membrane rafts and the TLR4-raft association is stimulated by LPS (Table 1). Such results have been obtained in studies based on density gradient centrifugation of Triton X-100 cell lysates yielding detergent-resistant membrane (DRM) fraction [29, 31, 69]. This approach is based on model membrane studies indicating that regions of the plasma membrane rich in saturated lipids and cholesterol are insoluble in non-ionic detergents, like Triton X-100, due to the tight packing of the lipids [70]. DRM fragments can be subsequently separated from other membrane and cytosol components based on their low density due to high lipid content. Ample studies have indicated that the protein and lipid composition of the DRM fraction isolated in density gradients is variable and depends on the protocol used, in particular the detergent type and its concentration, temperature, and duration of cell solubilization [71–73]. One possible reason of this DRM variability is selective extraction of proteins depending on the strength of their association with native rafts in the membrane or, conversely, incorporation of non-raft proteins during membrane solubilization [74, 75]. Thus, although isolation and characterization of the DRM fraction is a useful approach for raft analysis, it should be born in mind that the DRM fraction is not identical with rafts of the intact plasma membrane. It has been hypothesized, however, that the different composition of isolated DRMs can actually reflect the inherent variability of protein concentration affecting lipid order in native plasma membrane rafts [49].

**Table 1:** Studies supporting the involvement of plasma membrane rafts in TLR4-triggered signaling

Proteins and lipids detected in rafts	LPS chemotype	Cells	Technique	Lipid related events, comments	References
Constitutively present: CD14, CD55, CD47, CD32, CD64; After LPS stimulation: TLR4, CD11b/CD18, CD16a CD36, CD81; exclusion of CD47	LPS from *Salmonella minnesota* ^a^	Human blood monocytes	FRET between CD14 and the other proteins; Sucrose gradient centrifugation of 0.5 % TX-100 cell lysates	Application of propyl-CD or nystatin reduces CD14/CD11b co-localization induced by LPS; Exogenous long-chain ceramide induces co-clustering of CD14 with other cell surface receptors with exception of TLR4	[29]
Constitutively present: CD14, GM1, Hsp70, Hsp90; After LPS stimulation: TLR4, GDF5, CXCR4, MyD88, JNK	ReLPS 595 from *Salmonella minnesota; * ReLPS from *Escherichia coli*	MonoMac-6 (mature human monocyte cell line)^b^; CHO expressing human CD14 and human TLR4^c^; Human blood monocytes^c,d^	Sucrose gradient centrifugation of 1 % TX-100 cell lysates; FRET between GM1 and indicated proteins	Application of mβCD or nystatin inhibits association of CD14, TLR4, Hsp70, Hsp90, CXCR4 with raft fractions and inhibits TNF-α production	[29, 31, 190]
After LPS stimulation: TLR4	ReLPS 595 from *Salmonella minnesota*	Human blood monocytes	FRAP	Application of mβCD prevents immobilization of TLR4 in the plasma membrane induced by LPS	[31, 77]^e^
Constitutively present: CD14, flotillin, CD55; After LPS stimulation: active Cdc42 and p38; exclusion of Rac	sLPS from *Escherichia coli* 0111:B4	Human blood neutrophils	Sucrose gradient centrifugation of 1 % TX-100 cell lysates	Application of mβCD upregulates Cdc42 and p38 activity and induces actin polymerization but inhibits subsequent LPS-induced Cdc42 and p38 activation	[200]
After LPS stimulation: CD14, ERK, p38	LPS^a^	Raw264.7	Sucrose gradient centrifugation of 1 % TX-100 cell lysates	mβCD and nystatin do not inhibit TNF-α production and ERK activation induced by LPS but have stimulatory effect themselves	[117]
Constitutively present: CD14, flotillin-1, GM1, small amounts of CD9, CD81; After LPS stimulation: TLR4 (small amounts), enrichment of CD14, CD9, CD81	sLPS from *Escherichia coli* 055:B5	Bone marrow-derived macrophages of mice	Sucrose gradient centrifugation of 1 % TX-100 cell lysates	Application of propyl-CD inhibits TNF-α induced by LPS	[201]
Constitutively present, enriched after LPS stimulation: CD14, Hsp70, Hsp90, Lyn, Hck, Fgr, CD44, acid sphingomyelinase, gp91 (phox); After LPS stimulation: several proteins involved in protein ubiquitination, proteasome subunits, active (phosphorylated) ERK and MEK; Lack of TLR4	sLPS from *Escherichia coli* 0111:B4	Raw264.7	Sucrose gradient centrifugation of 1 % TX-100 cell lysates, proteomic analysis of gradient fractions	Proteasome-mediated regulation of ERK activity in raft fractions mβCD induces ERK activation but attenuates subsequent LPS-induced activation of the kinase. Nystatin also inhibits ERK activation in LPS-stimulated cells	[84]
Constitutively present: CD14; After LPS stimulation: TLR4, Hsp70	sLPS from *Escherichia coli* 0111:B4	THP1 monocytes differentiated by PMA	Sucrose gradient centrifugation of 1 % TX-100 cell lysates	Imipramine used as ASMase inhibitor diminishes TLR4 and Hsp70 recruitment to rafts, ERK, p38 and JNK activation and TNF-α production induced by LPS. All the inhibitory events reversed by exogenous C_2_-ceramide	[83]
Constitutively present: CD14, flotillin-1, caveolin-1, TLR4	LPS^a^ from *Salmonella typhimurium*	Peritonel macrophages and bone marrow-derived macrophages of *Abca1* ^−*M/*−*M*^ mice	Optiprep gradient centrifugation of sonicated membrane fraction (no detergent used)	ABCA1 deficiency increases partition of TLR4 to raft fraction, activity of NFκB and MAP kinases, production of pro-inflammatory cytokines in LPS-stimulated cells; mβCD decreases TNF-α, IL-6 and IL-12p40 production	[80, 202]
Enriched after LPS stimulation: TLR4, TRIF, MyD88	sLPS from *Escherichia coli* 0111:B4	Raw264.7; HEK293T and Ba/F3 (interleukin-3-dependent murine pro-3 cell line) expressing TLR/MD-2	Sucrose gradient centrifugation of 1–1.5 % TX-100 cell lysates; Co-localization of TLR4 and GM1	Lauric acid mimicks LPS inducing dimerization of TLR4 in raft fractions; DHA inhibits LPS- and lauric acid-induced association of TLR4 with rafts; Nystatin and mβCD decrease dimerization of TLR4 in raft fraction, prevents NFκB activation and target gene expression induced by LPS and lauric acid	[81, 82]

*Propyl-CD* 2-hydroxypropyl-β-cyclodextrin, *mβCD* methyl-β-cyclodextrin, *TX-100* Triton X-100, *Abca1* ^−*M/*−*M*^ homozygous macrophage-specific ATP-binding cassette transporter A1 knockout mice

^a^Origin or chemotype of LPS not specified

^b^Cells used for DRM isolation

^c^Cells used for FRET studies

^d^Cells used to analyze TNF-α production

^e^Similar data shown on TLR2-raft association stimulated by lipoteichoic acid

Due to technical limitations of detergent-based biochemical approaches, microscopic techniques are especially useful for examining native rafts in living cells. In recent years, studies on rafts have benefited exceptionally from the development of super-resolution microscopy [76] which is yet to be employed to studies on the involvement of plasma membrane rafts in LPS-triggered signaling. Thus far, in support of the biochemical data, measurements of the fluorescence resonance energy transfer (FRET) between the constitutive raft components GM1 or CD14 and selected plasma membrane proteins have indicated that LPS induces the assembly of a raft-associated multimolecular complex composed of TLR4 and other proteins potentially involved in LPS recognition [29, 69]. In line with these data, LPS has been shown to reduce the lateral mobility of TLR4 in the plane of the plasma membrane. This LPS-induced confined diffusion of TLR4, revealed by measurements of fluorescence recovery after photobleaching (FRAP), has been ascribed to TLR4 trapping within plasma membrane rafts [31, 77].

Taking into account that raft assembly is driven by interactions of sphingolipids and cholesterol, the observed disturbances in TLR4 signaling following changes of the cellular level of these lipids favor the idea of raft involvement in LPS-induced inflammatory responses (Table 1). Extraction or sequestration of cholesterol with cyclodextrin or nystatin has been shown to disturb clustering of TLR4 and accessory proteins in rafts and to inhibit LPS-induced TNF-α production [29, 69, 77]. On the other hand, a deficiency of ATP-binding cassette transporters A1 or G1, linked with cholesterol elevation and an apparent increase of raft content in macrophages, enhanced the partition of TLR4 to raft fractions and augmented pro-inflammatory signaling [78–80]. Similarly, the pro-inflammatory effect of an exposure of RAW264 cells to saturated fatty acids [81, 82] can be interpreted as a result of enhanced raft assembly.

It is noteworthy that the majority of data supporting LPS-induced accumulation of TLR4 in rafts and the assembly of a raft-based multimolecular complex containing TLR4 were obtained by microscopic and biochemical studies of monocytes and cells of established monocyte lines [29, 31, 69, 83]. In contrast, a recent proteomic analysis of the DRM fraction isolated from RAW264 macrophage-like cells indicated a lack of TLR4 in this raft-derived fraction, regardless of LPS stimulation [84]. Those data suggest that in macrophages the association of TLR4 with rafts can be dynamic and/or too weak to allow its preservation during fractionation of Triton X-100 cell lysates over density gradients. In accordance, studies on the co-localization of TLR4 and CD14 in J774 macrophage-like cells showed that the proteins co-localized transiently and their coincidence was confined to lamellae of LPS-stimulated cells [85]. Despite the failure to detect TLR4 in the DRM fraction of RAW264 cells, the proteomic analysis identified several dozen proteins which were either enriched or recruited to this fraction after 5 and 30 min of LPS stimulation [84]. The list of proteins enriched in the DRM after LPS stimulation includes CD14, CD44, Src family tyrosine kinases Lyn, Hck, and Fgr, Hsp70, Hsp90, acid sphingomyelinase, and NADPH oxidase subunit gp91^*phox*^, supporting other ample data on the involvement of these proteins in TLR4 signaling.

## Participation of CD14 in LPS binding and signal transduction

### CD14 is more than LPS-binding protein

The role of CD14 as a key component of LPS-induced inflammatory responses was indicated by studies on transgenic mice expressing human CD14 and mice deficient in CD14. The transgenic mice were hypersensitive to LPS while mice devoid of CD14 did not develop septic shock or accumulate pro-inflammatory cytokines in the blood following exposure to *E. coli* or intraperitoneal injection of LPS at a dose lethal to wild-type mice [86–88]. This protective effect of CD14 deficiency reflects a fatal role of this protein in exaggerating the inflammatory response in the course of systemic septic shock, although during local infection, the CD14 involvement in combating invading bacteria can be beneficial, as discussed by Zanoni and Granucci [89].

CD14 has long been considered mainly as a molecule which concentrates and delivers LPS to TLR4/MD-2 facilitating TLR4 activation [17, 90]. Originally, however, CD14 was envisioned as a pattern recognition receptor [91], but a lack of a transmembrane and cytoplasmic domain called the signaling role of CD14 into question. The importance of the membrane localization of CD14 for LPS-induced signaling was also negated by the fact that a soluble CD14 (sCD14) devoid of the GPI moiety could substitute for membrane CD14 (mCD14) endowing cells that normally do not express CD14 or express it at a very low level, like endothelial and epithelial cells, as well as *Cd14* ^−*/*−^ macrophages, with the ability to produce TNF-α and some other pro-inflammatory cytokines in response to LPS stimulation [87, 92, 93].

**Fig. 2:**
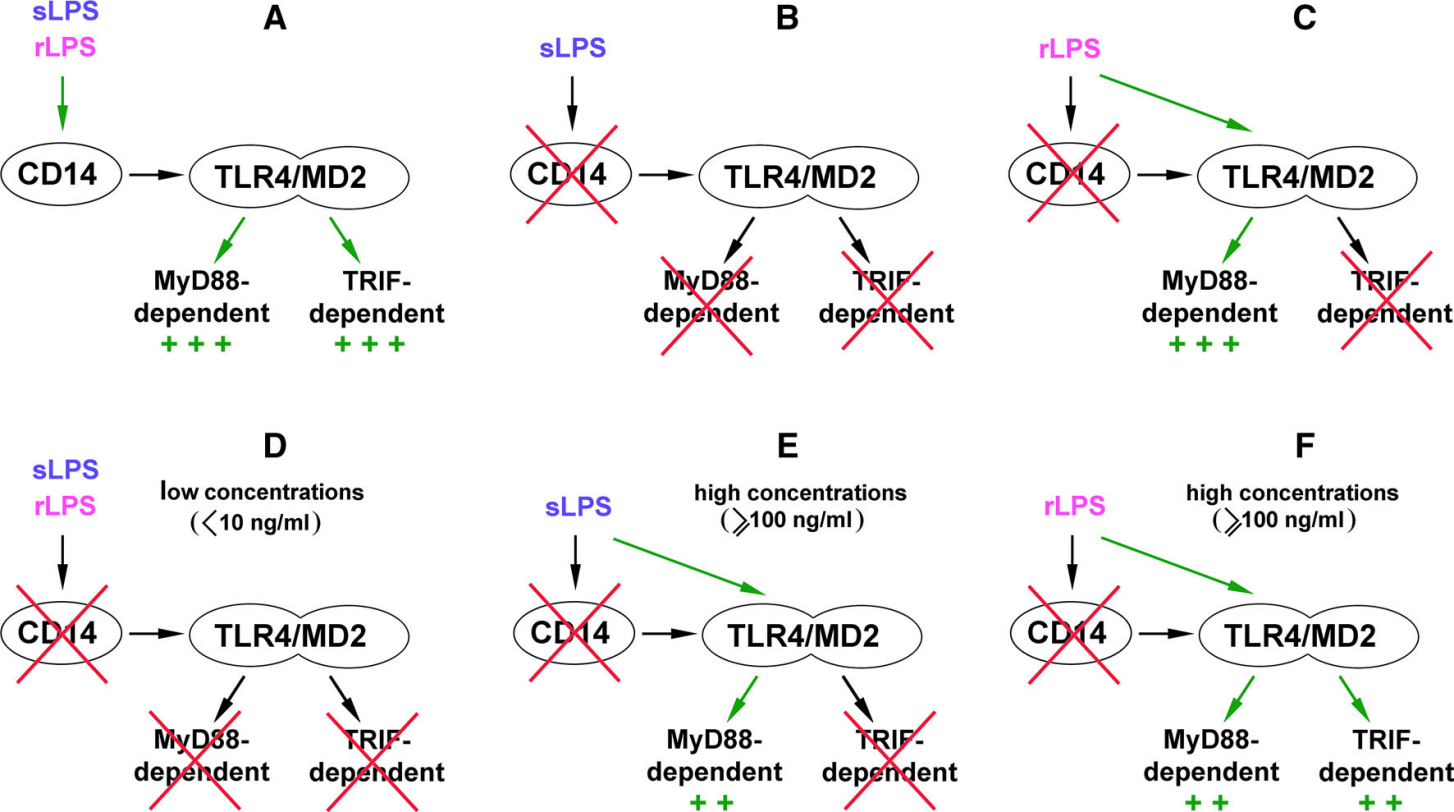
Participation of CD14 in TLR4 signaling pathways triggered by sLPS and rLPS. **a** In the presence of CD14, sLPS and rLPS activate TLR4 and trigger MyD88- and TRIF-dependent pathways with similar intensity. **b**, **c** Studies performed on macrophages bearing the Heedless mutation of *Cd14* have indicated that sLPS requires CD14 to activate TLR4 (**b**), while rLPS can induce TRIF-dependent signaling of TLR4 in CD14-deficient cells (**c**). **d**–**f** Other studies suggest that the requirement of CD14 for activation of TLR4 varies depending on the concentration of the endotoxin. At low concentrations, sLPS or rLPS are unable to activate TLR4 without the involvement of CD14 (**d**). At higher doses of sLPS, CD14 is dispensable for initiation of MyD88-dependent pathway of TLR4, although the production of TNF-α is submaximal in these conditions (**e**). When present in relatively high concentrations, rLPS can induce submaximal activation of both signaling pathways of TLR4 without binding to CD14 (**f**)

Indications of a more complex role of CD14 in LPS-induced responses have come from a series of studies performed by the Goyert’s and Beutler’s groups. The former one found that some genes, like *IP*-*10*, that are now known to be TRIF-dependent, were minimally expressed in macrophages isolated from *Cd14* ^−*/*−^ mice and stimulated with K235 LPS of *E. coli*. Simultaneously, expression of genes encoding TNF-α and IL-1β could be induced in a CD14-independent manner [94]. Studies of Beutler and co-authors have unraveled a general picture of a disparate requirement for CD14 to trigger the MyD88-dependent and TRIF-dependent signaling pathways of TLR4. In addition, the involvement of CD14 was found to differently determine TLR4 responses to so-called smooth (s) and rough (r) chemotypes of LPS [88]. rLPS is produced by some Gram-negative bacteria, especially *Enterobacteriaceae* with mutations in genes involved in the O-chain synthesis. Therefore, it is devoid of the *O*-polysaccharide chain and can bear incomplete core oligosaccharides in contrast to the sugar-linked smooth (s) LPS produced, e.g., by most *E. coli* strains including the K235 strain. In studies on sLPS and rLPS signaling requirements, *N*-ethyl-*N*-nitrosourea-mutated mice were used bearing a recessive Heedless mutation identified as a premature stop codon in *Cd14* gene [88]. The lack of CD14 expression abolished the TRIF-dependent pathway regardless of the LPS chemotype used for cell stimulation. Thus, macrophages isolated from Heedless homozygotes failed to produce type I IFN as a consequence of a lack of IRF3 activation and did not display induction of IFN-inducible genes in response to sLPS or lipid A. Accordingly, no type I IFN was found in the blood of Heedless mutant mice injected with sLPS or rLPS. In summary, both sLPS and rLPS seemed to share a common requirement for CD14 participation in triggering TRIF-dependent signaling (Fig. 2a–c). This important finding was addressed in further studies on the CD14 signaling abilities discussed below.

There was, however, a clear distinction between the ability of sLPS and rLPS to induce TNF-α production in the absence of CD14. Both mice and ex vivo macrophages bearing the Heedless mutation failed to produce TNF-α in response to sLPS but retained the ability to generate TNF-α as a result of the activation of NFκB and MAP kinases after exposure to rLPS or lipid A. The data suggested that MyD88-dependent signaling can be generated by TLR4/MD-2 alone in response to rLPS but triggering this signaling pathway by sLPS requires CD14 (Fig. 2b, c). Such a strict requirement of CD14 for sLPS-induced TNF-α production can be typical for lower doses of sLPS, as parallel ex vivo studies performed on *Cd14* ^−*/*−^ macrophages by the Goyert’s group indicated that at concentrations equal or higher than 100 ng/ml sLPS displayed some potency for TNF-α induction. This potency was higher for rLPS [95]. Although both groups interpreted their data differently, both sets of results in fact seem to indicate that CD14 participation ameliorates the differences between the ability of rLPS and sLPS to activate TLR4. This assumption is supported by recent in vivo studies in which rLPS and sLPS elicited nearly similar accumulation of pro-inflammatory cytokines in the serum of mice injected with 1 mg/ml of the endotoxin [96]. These data leave unresolved the question of the meaning of the ability of rLPS to activate cells in a CD14-independent manner. It is conceivable that rLPS can induce production of pro-inflammatory cytokines in CD14-negative cells which otherwise can benefit also from the assistance of sCD14, as discussed above. In agreement with this assumption, it was found that mast cells, which do not express CD14, produce IL-6 when stimulated with rLPS but not sLPS [93].

Subsequent studies performed on RAW264 cells exposed to antibodies blocking the LPS binding to CD14 showed that the requirement for CD14 participation in LPS-induced signaling vary depending on the concentration of both sLPS and rLPS. At low doses of LPS (<10 ng/ml), CD14 is crucial for the production of TNF-α and RANTES (used to gauge MyD88- and TRIF-dependent signaling, respectively) induced by either LPS chemotype [97] (Fig. 2d). These data are consistent with earlier findings showing that participation of CD14 markedly increases responsiveness of cells to low concentrations of rLPS or sLPS [90, 95]. At higher doses, sLPS induces moderate production of TNF-α also without the CD14 participation (Fig. 2e), resembling results of earlier studies of the Goyert’s group [94, 95]. Notably, rLPS in these conditions activates the moderate production of both TNF-α and RANTES (Fig. 2f). In addition, an assistance of LBP was indispensable to induce maximal generation of these cytokines in response to sLPS but not to higher doses of rLPS [97, 98]. In summary, it is clear that rLPS relies on CD14 assistance to a lower extent than does sLPS in activating TLR4, and of the two signaling pathways triggered by TLR4, the involvement of CD14 is especially important for the TRIF-dependent one.

Questions arise as to the molecular mechanism of CD14 (and LBP)-independent activation of cells by LPS. In the absence of CD14, albumin can bind LPS monomers without the assistance of LBP and deliver them to TLR4/MD-2 [99]. Alternatively, LPS could be incorporated into rafts of the plasma membrane and subsequently bind to the receptor complex. The membrane incorporation could be facilitated by the lack of the O-chain and thus higher hydrophobicity of rLPS in comparison with sLPS [93]. Integration of LPS into the plasma membrane could also be potentiated by the aggregated state of rLPS [100]. The incorporation of rLPS into membranes can induce profound changes of the membrane raft organization, as revealed by studies on the interaction of ReLPS (the shortest form of rLPS also used in [88, 97]) with model membranes performed by solid-state NMR spectroscopy. When mixed with DEPE/sphingomyelin/cholesterol liposomes, a ternary lipid mixture in which the sphingomyelin/cholesterol-rich *L*_o_ phase co-exists with the DEPE-rich *L*_d_ phase, ReLPS induced coalescence and expansion of the *L*_o_ phase [101]. These data correspond with the results of microscopic studies on LPS organization in giant liposomes composed of polar lipids isolated from *E. coli*. In these membranes, rLPS formed micron-sized gel-like microdomains while sLPS formed small clusters about 380 nm in diameter [102]. A line of other studies performed on model membrane also indicated that sLPS can spontaneously incorporate into membranes. The preferred sites of sLPS binding and incorporation were sphingomyelin/cholesterol-rich domains formed in DOPC/sphingomyelin/cholesterol liposomes [103, 104]. These findings suggest the importance of a direct interaction of rLPS and sLPS with plasma membrane rafts. In the case of rLPS, its incorporation could efficiently induce coalescence of rafts, possibly inducing TLR4 dimerization and pro-inflammatory signaling. The coalescence of nanoscale rafts into more stable, larger raft domains is fundamental for their functioning in the plasma membrane. It causes co-patching of raft proteins and lipids into functional signaling platforms with simultaneous exclusion of non-raft proteins [42].

It should be noted that in model membrane studies discussed above relatively high LPS concentrations (e.g., in the range of μg/ml [103]) were used to reveal changes of membrane organization. Stimulation of cells with high doses of LPS could also facilitate a direct action of LPS, particularly rLPS, on the plasma membrane. This assumption could explain the activation of TRIF signaling by 100–1,000 ng/ml rLPS in RAW264 cells even when the binding of rLPS to CD14 was inhibited, and the lack of such activation by 10–100 ng/ml rLPS or lipid A in CD14-deficient macrophages bearing the Heedless mutation [88, 97] (Fig. 2c vs. f). These data suggest that, when present in higher concentrations, rLPS can trigger TRIF-dependent signaling bypassing the requirement for the binding to CD14 which is otherwise required for the internalization of TLR4/LPS leading to TRIF recruitment, as discussed in the next section.

### CD14 participates in internalization of TLR4/LPS

Both sLPS and, to a lower extent, rLPS rely on CD14 assistance to activate the TRIF-dependent pathway of TLR4 (Fig. 2). Why is the CD14 participation required for activation of this pathway?

The importance of CD14 for the initiation of the TRIF-dependent pathway has been linked with internalization of TLR4 which is essential for this signaling cascade. The link between the endocytosis of LPS-activated TLR4 and the subsequent TRIF-mediated signaling has been shown by a line of data. It has been demonstrated that the surface level of the receptor decreases in LPS-stimulated macrophages, monocytes, and CD14/TLR4/MD-2-transfected Ba/F3 cells. TLR4 co-localized with markers of early/sorting endosomes and its clearance from the cell surface was inhibited by dynasore, an inhibitor of the GTPase dynamin controlling the pinching-off of endocytic vesicles [26, 27, 105]. Concomitantly, dynasore abolished the TRIF-dependent signaling indicating that endocytosis of TLR4/LPS is important for this signaling pathway of TLR4 [27, 36]. The endocytosis of TLR4 is supposed to follow MyD88-dependent signaling originating from the plasma membrane [27, 105, 106] and the switch from the plasma membrane MyD88-based to the TRIF-dependent endosomal signaling of TLR4 could be controlled by phosphatidylinositol 4,5-bisphosphate [PI(4,5)P_2_] turnover in the plasma membrane. The drop of PI(4,5)P_2_ level in the membrane of endosomes was proposed to facilitate the disassembly of the TIRAP/MyD88 signaling complex and association of TRAM/TRIF adaptors [27, 107]. In support of this thesis, the MyD88-dependent activation of NFκB was enhanced in HEK293-CD14/TLR4/MD-2 transfectants when the endocytosis of TLR4 was disrupted [26]. Overaccumulation of PI(4,5)P_2_ in dendritic cells as a result of inactivation of the 110δ isoform of class I phosphatidylinositol 3-kinase (PI3-kinase), which phosphorylates PI(4,5)P_2_ to phosphatidylinositol 3,4,5-trisphosphate [PI(3,4,5)P_3_], was one of the means of achieving this goal [106].

**Fig. 3:**
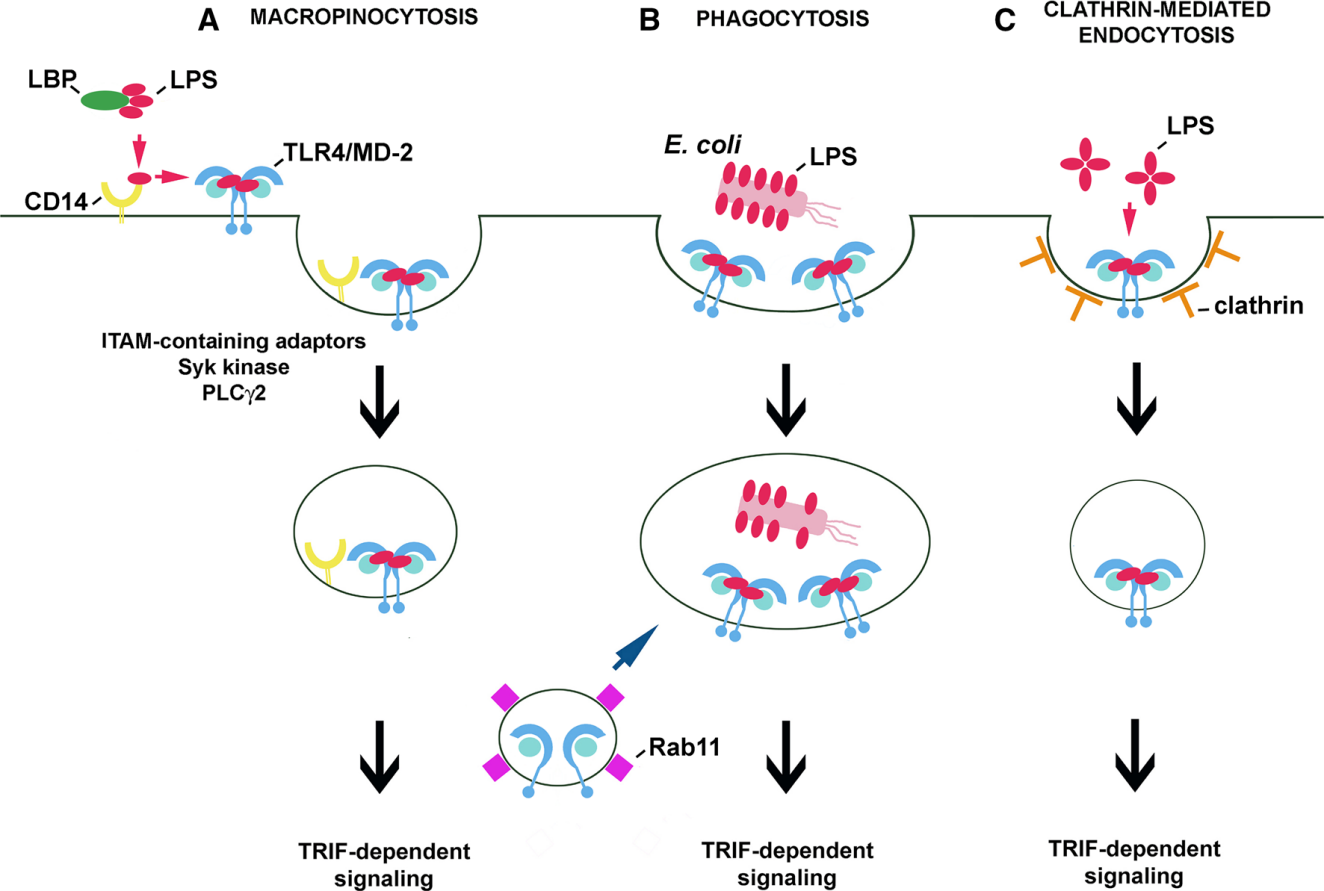
Routes of internalization of LPS-activated TLR4/MD-2. Internalization of TLR4/MD-2 can be induced in a CD14-dependent (**a**) or CD14-independent manner (**b**, **c**). **a** When LPS monomers are bound by CD14, the protein transfers the LPS to the TLR4/MD-2 complex which, after inducing the MyD88-dependent pathway (not shown), undergoes macropinocytosis controlled by CD14 and required for TRIF-dependent signaling. Similar steps of TLR4 activation take place during phagocytosis of *E. coli* in CD14-expressing cells. **b** In dendritic cells, phagocytosis of *E. coli* can bypass the requirement for the involvement of CD14 in TLR4/MD-2 uptake and activation. The intracellular pool of TLR4/MD-2 located in Rab11-containing recycling endosomes can be delivered to phagosomes containing *E. coli* and possibly to endosomes during macropinocytosis. **c** TLR4/MD-2/LPS complexes can also undergo clathrin-mediated endocytosis, although the initial steps of this process are unclear

A line of data indicates that CD14 controls the internalization of LPS-activated TLR4. Indeed, studies performed on bone marrow-derived macrophages and dendritic cells confirmed a significant time-dependent decrease of the cell surface level of TLR4 in wild type but not in *Cd14* ^−*/*−^ cells exposed to 1 μg/ml sLPS [28]. Simultaneously, the CD14-deficient cells failed to trigger TRIF-dependent signaling in response to sLPS, in agreement with results of earlier studies discussed above [88, 94, 97]. It has been established that the clearance of CD14 and TLR4 from the cell surface, and subsequent IRF3 activation, and type I IFN production all require the activity of Syk kinase. Syk binds to proteins containing the immunoreceptor tyrosine-based activation motif (ITAM) and contributes to the downstream activation of phospholipase Cγ2 (PLCγ2) [28]. Furthermore, PLCγ2, but not PLCγ1, was found to account for inositol trisphosphate (IP_3_) generation and subsequent release of Ca^2+^ from intracellular stores required for TLR4 endocytosis and IRF3 activation in RAW264 cells and bone marrow-derived murine macrophages [108]. These data support the idea that CD14 controls TLR4/LPS macropinocytosis by activating ITAM-mediated events which lead to Syk-PLCγ2-dependent internalization of TLR4 [28] (Fig. 3a). This proposed chain of events is independent of the Src kinase-PLCγ2 axis found to control NFAT activation in dendritic cells only [109, 110]. The data on TLR4 endocytosis confirmed a biphasic scenario of TLR4 activation with the second phase being dependent on the CD14-controlled internalization of the LPS/CD14/TLR4 complex. It is noteworthy that macropinocytosis of LPS/CD14/TLR4 can overlap the CD14- and SR-A-mediated uptake of LPS leading to is detoxification. This non-signaling uptake of LPS does not involve TLR4 and in fact can compete with the TLR4/LPS uptake which contributes to TLR4 signaling [36, 111].

The endosomal origin of the TRIF-dependent signaling of TLR4 has been verified by the effects exerted on the signaling by phagocytosis of *E. coli* by macrophages and dendritic cells. The phagocytosis was expected to bypass the lack of CD14 in knockout cells, force TLR4 internalization and eventually induce generation of the TRIF-dependent cascade. The prediction turned out to be correct for dendritic cells only. In CD14-deficient macrophages, the phagocytosis of *E. coli* failed to restore endocytosis of TLR4 and TRIF signaling [28]. The authors ascribed those contrasting results to a more “permissive” nature of dendritic cells facilitating TLR4 uptake in the absence of CD14 (Fig. 3b).

Some studies questioned the need of TLR4 internalization for triggering the TRIF-dependent pathway during phagocytosis of *E. coli* and instead ascribed the signaling abilities to an intracellular pool of TLR4. This pool of TLR4 resides in Rab11-positive recycling endosomes and can be transported to the *E. coli*-containing phagosomes, thereby triggering the TRIF-dependent pathway. The authors suggested that the internal pool of TLR4 can induce the TRIF-dependent cascade without previously being engaged in MyD88-dependent signaling in the plasma membrane, provided LPS reaches the endosomal compartment [112]. These data do not explain why the phagocytosis of *E. coli* by *Cd14* ^−*/*−^ macrophages, which proceeded without concomitant TLR4 uptake, did not restore TRIF-dependent signaling [28] thereby suggesting that TLR4 delivered to phagosomes from Rab11-bearing endosomes can only amplify TRIF-dependent signaling triggered by internalized TLR4.

Beside phagocytosis of *E. coli* in dendritic cells, one more CD14-independent pathway of TLR4/LPS uptake leading to TRIF activation can be considered. It has recently been found that endocytosis of LPS-containing liposomes can proceed unassisted by CD14 in clathrin-coated vesicles (Fig. 3c). Endocytosis of LPS liposomes led to IRF3 activation and RANTES production in macrophages without a concomitant TNF-α and IL-6 release [113]. Such endocytic pathway could also explain the CD14-independent activation of RANTES production induced at higher doses of rLPS (see Fig. 2f). An involvement of clathrin-coated endocytosis in the internalization of LPS and TLR4/LPS was indicated by electron microscopy and immunofluorescence studies [26, 114]. About ten times more LPS is internalized in non-coated than in coated vesicles [114], yet drugs inferring with clathrin-dependent endocytosis significantly inhibit TRIF-dependent signaling in LPS-stimulated cells [36, 115]. Further studies are required to reveal whether CD14 affects clathrin-mediated uptake of TLR4/LPS, which factors direct TLR4/LPS for macropinocytosis or clathrin-mediated endocytosis, and whether the pathway of TLR4 internalization modulates its signaling, as suggested for receptor tyrosine kinases [116].

### Is raft localization of CD14 crucial for its involvement in LPS-triggered events?

The relevance of the raft association of CD14 to its involvement in TLR4 signaling in monocytes/macrophages, key players in inflammatory reaction, has long puzzled researchers. Circumstantial evidence supporting such dependence came from cell fractionation studies showing enrichment of CD14 in the raft-originating DRM fraction of LPS-stimulated RAW264 cells [84, 117]. A more direct approach to this issue relied on a comparison of cell activation by LPS mediated by GPI-anchored and chimeric transmembrane forms of CD14. For this purpose, CD14 was fused to the transmembrane plus cytoplasmic fragment of either LDL receptor or tissue factor and expressed in THP-1 cells [114, 118, 119]. The CD14-tissue factor chimera localized to the Triton X-100-soluble fraction, yet it induced NFκB activation, p38 phosphorylation and IL-8 and TNF-α production in a similar manner as its wild-type raft-anchored counterpart [118]. Of note, the MyD88- and TRIF-dependent signaling could not be distinguished at the time when those studies were carried out. It seems, therefore, that the apparent dispensability of GPI anchoring for CD14 functioning can be revisited in view of our present understanding of the distinct requirements for the involvement of CD14 in the two signaling pathways of TLR4.

Raft localization of CD14 can be critical for macropinocytosis of LPS-activated TLR4. The CD14-mediated macropinocytosis of the LPS/CD14/TLR4 complex relies on the formation of large non-coated vesicles and involves Syk kinase activity, as described above [28]. A recent discovery of Syk-mediated internalization of CD36, a scavenger receptor localized in rafts (and caveolae), has paved the way for consideration of how CD14 could trigger the LPS/CD14/TLR4 internalization in macrophages. Similarly to CD36, CD14 could facilitate the assembly of a multimolecular complex composed of tetraspanins, β1 and β2 integrins, and ITAM-bearing Fcγ receptors [120]. Notably, FRET analysis indicated that tetraspanin CD81, integrin β2 (CD11/CD18) and Fcγ receptors gather in the vicinity of CD14 and TLR4 in LPS-stimulated human monocytes [29, 31, 121] (Table 1). Subsequent binding of Syk to ITAMs, both likely to be phosphorylated by Src family kinases, would then trigger a cascade of phosphorylations of a variety of adaptor and scaffolding proteins, and recruitment of lipid kinases and PLCγ2 leading to the activation of Rho GTPases and WASP/Scar proteins. This chain of events could control local actin polymerization providing the driving force for internalization of LPS/CD14/TLR4, as has been deciphered for Fcγ receptor-mediated phagocytosis. Taking into consideration that Fcγ receptors are functionally connected with TLR4 and associate with rafts in LPS-stimulated cells [29, 121, 122] and that phosphorylation of their ITAMs by Src family kinases in the rafts is well established [64, 65], the CD14-bearing rafts would be preferred sites of the assembly of the multimolecular complex mediating the internalization of LPS/CD14/TLR4. Induction of raft reorganization by high doses of rLPS, as found in model membrane studies [101], could trigger a similar chain of events leading to TLR4 internalization without prior binding of rLPS to CD14 (see Fig. 2f). A potential caveat of this model that needs to be addressed is the postulated lack of an involvement of Src family kinases in TLR4 internalization inferred from the application of Src inhibitor-1 [28].

In addition to the CD14-controlled macropinocytosis of LPS-activated TLR4, another role is ascribed to CD14 exclusively in dendritic cells. The CD14-dependent NFAT activation in these cells provides a most clear indication that the raft localization of CD14 is critical for its functioning in LPS-stimulated cells. Upon LPS stimulation of dendritic cells CD14 triggers an influx of Ca^2+^ leading to calcineurin-mediated activation of NFAT independently of TLR4. Eventually, production of IL-2, prostaglandin E_2_ as well as apoptosis of the cells occurs [109, 110]. To fulfill this function, CD14 needs to be membrane anchored as sCD14 does not support NFAT activation. It was found that mCD14 activates Src kinases and PLCγ2 leading to IP_3_ generation and an influx of extracellular Ca^2+^ to the cytoplasm. Cholesterol depletion abolished this Ca^2+^ signaling, suggesting that raft integrity is crucial for the co-operation of CD14 with raft-anchored Src kinases [109].

## Involvement of Lyn in LPS-induced TLR4 signaling pathways

### Tyrosine kinases of the Src family in LPS-induced signaling

Toll-like receptor 4 signaling relies on cascades of protein serine–threonine phosphorylation and polyubiquitination events. Activation of this receptor also triggers protein tyrosine phosphorylation catalyzed by multiple protein tyrosine kinases including Bruton’s tyrosine kinase [123, 124], Syk kinase [28, 125], and kinases of the Src family [126–128]. Of note, the activity of all these kinases is crucial for the signaling by raft-associated receptors. The most thoroughly characterized examples of that come from studies on the involvement of Src family kinases and Syk kinase in the signaling cascades of receptors containing ITAM signaling motifs, e.g., FcγRIIa, FcεRI, and TCR [64, 65, 129].

In terms of TLR4 activation, pretreatment of human monocytes and macrophages with herbimycin A or genistein, broad-spectrum inhibitors of tyrosine kinases, or with PP1, an inhibitor of Src family kinases, reduced LPS-induced production of several cytokines like TNF-α, IL-1α, IL-6, IL-10, and IP-10, and prevented the activation of MAP kinases and NFκB [126, 127, 130, 131]. In contrast, macrophages isolated form *hck* ^−*/*−^ *fgr* ^−*/*−^ *lyn* ^−*/*−^ triple knockout mice released normal or even increased amounts of TNF-α, IL-1, IL-6, and NO and showed no impairment of the activation of MAP kinases and NFκB [132]. Double-deficient *hck* ^−*/*−^ *fgr* ^−*/*−^ mice displayed an increased resistance to endotoxic shock which was ascribed, however, to defective integrin signaling and consequent reduced neutrophil migration into the tissue rather than to a direct effect of the lack of Hck and Fgr activities on cytokine production [133]. The discrepancies between the effects of drug application and the knockout of the tyrosine kinase genes on the pro-inflammatory reaction of cells are likely to be due to the fact that the Src family of protein tyrosine kinases comprises nine members: Src, Lyn, Hck, Fgr, Fyn, Yes, Lck, Ylk, and Blk, of which the first six are known to be expressed in macrophages [131]. All these kinases share a common domain structure, with the N-terminal domain undergoing myristoylation and palmitoylation, the latter facilitating anchoring of the kinase in plasma membrane rafts. The kinases also contain the SH3 and SH2 domains, the catalytic domain, and a short C-terminal tail controlling their conformation and enzymatic activity [134]. It has been suggested that the apparent failure to detect changes of LPS-induced responses in the knockout mice resulted from a compensation of the absence of some of the Src kinases by other family members. Indeed, short-term adenoviral overexpresion and siRNA knockdown studies have indicated that the Hck kinase activity controls the production of TNF-α and IL-6 induced by LPS in human macrophages. The kinase affects the activity of AP-1 transcription factor without influencing the activity of MAP kinases or NFκB [128]. LPS also triggers association of Hck with Vav, a Rho family guanine nucleotide exchange factor (RhoGEF) [135] involved in TNF-α production, as discussed below. In addition, Src kinase has been reported to act as a downstream effector of LPS-induced actin cytoskeleton rearrangements [136]. The participation of Lyn kinase in LPS-triggered TLR4 signaling is supported by the most extensive line of data.

### A positive role of Lyn in TLR4 signaling pathways

**Fig. 4:**
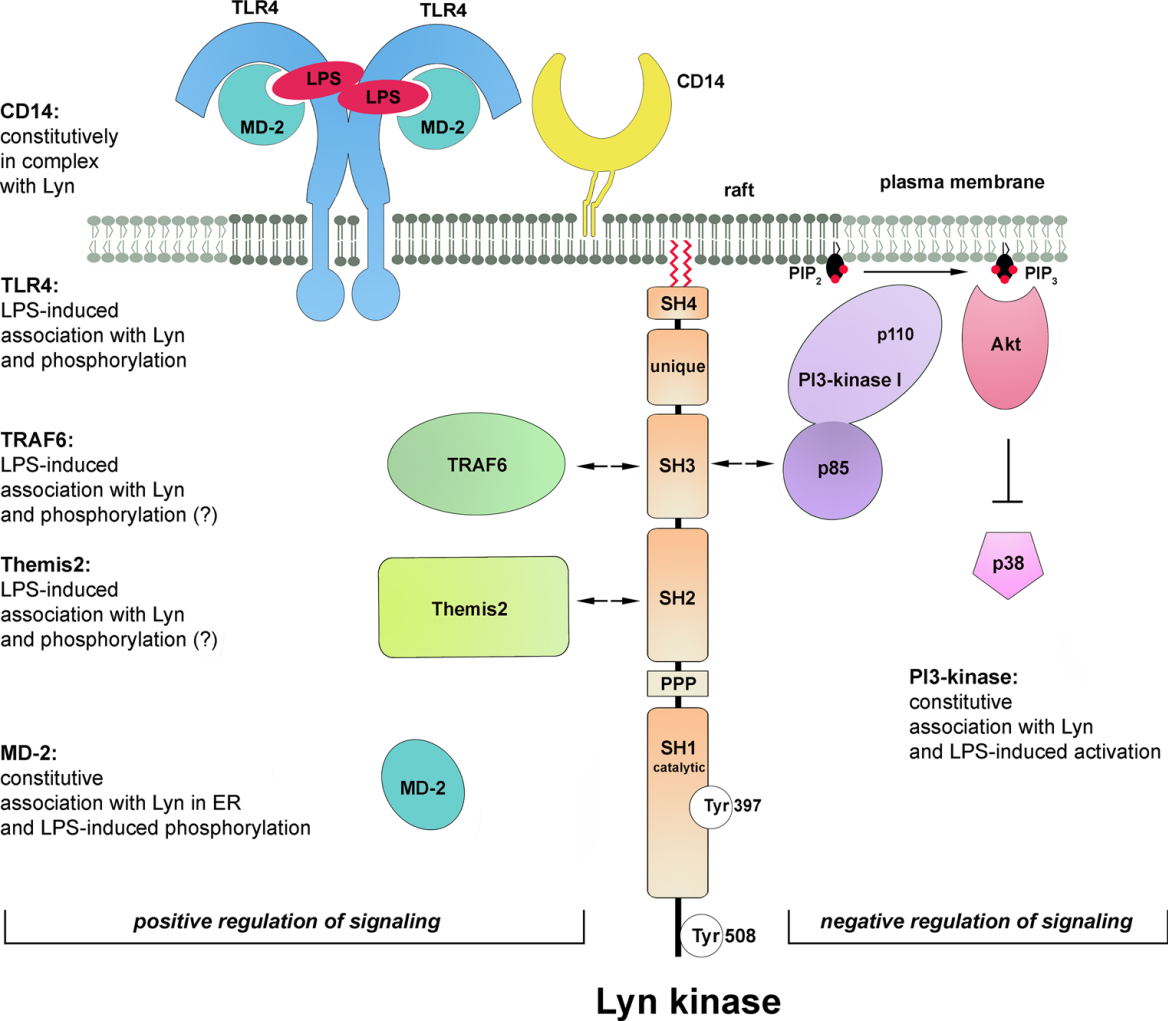
Participation of Lyn in TLR4 signaling. Lyn kinase either associates constitutively with proteins or associates with, and phosphorylates, indicated proteins after stimulation of cell with LPS. These events regulate positively or negatively TLR4-induced signaling (*left* and *right* side of the figure, respectively). *Arrows* indicate domains of Lyn most likely involved in binding of indicated proteins. The molecules are drawn not to scale

First indications on the involvement of Lyn kinase in LPS-induced signaling came from studies on CD14 protein. CD14 immunoprecipitated from human monocytes was found to be associated with Lyn kinase. The activity of the kinase increased shortly (1–5 min) after stimulation of the cells with 1 ng/ml sLPS [126]. Other studies on human monocytes revealed that within 1–5 min of stimulation with 10 ng/ml of ReLPS, TLR4 underwent tyrosine-phosphorylation [137]. Rapid Lyn activation was also observed in LPS- or taxol-treated mouse peritoneal macrophages [138] and more recently in human macrophages [128]. When expressed in HEK293 cells together with human TLR4 and MD-2, Lyn kinase co-immunoprecipitated with the receptor even in the absence of CD14. Recruitment of Lyn to TLR4 was triggered within 1 min of LPS stimulation of the cells with a maximal response at 15 min. Within the same time frame tyrosine phosphorylation of TLR4 was observed in the TLR4/MD-2-expressing HEK293 regardless of CD14 presence [127]. Those data suggested, although not proved, that Lyn kinase could be responsible for the phosphorylation of tyrosine reside(s) of TLR4. Crucially, further studies using HEK293 cells transfected with a constitutively active human CD4-TLR4 chimera indicated that TLR4 tyrosine phosphorylation was required for TLR4-induced signaling. Thus, mutation of tyrosine residues Y674A and Y680A in the TIR domain of TLR4 inhibited the recruitment of MyD88 and activation of IRAK-1 by the constitutively active form of the receptor. Furthermore, activation of NFκB, phosphorylation of p38 and JNK kinases, and RANTES production were also strongly suppressed, indicating that tyrosine phosphorylation of TLR4 is prerequisite for both the MyD88- and TRIF-dependent pathways. Similar suppression of TLR4 phosphorylation and signaling was found for P714H human and P712H murine TLR4, known as mutant receptors unresponsive to LPS [127]. Taken together, the data indicated the importance of protein tyrosine phosphorylation for TLR4 signaling and suggested a positive role of Lyn activity in this process (Fig. 4). The CD14/Lyn association and activation of CD14-associated kinase by LPS, found by Stefanova et al. [126], pointed to the importance of raft localization of Lyn in LPS-triggered signaling of TLR4. To our knowledge, however, this subject has not been addressed directly in any further studies.

In accordance with its plasma membrane localization, Lyn kinase was recently found to bind TRAF6 in response to LPS. TRAF6 is an E3 ubiquitin ligase which transiently associates with the myddosome and controls downstream steps of TLR4 signaling cascades affecting the TAK-1 kinase activity after catalyzing its own polyubiquitination via Lys^63^-linked chains [139]. The involvement of Lyn in LPS-induced TRAF6 activity has been verified with the use of mast cells from *lyn* ^−*/*−^ mice [140]. Remarkably, a low-level binding of TAK-1 to TRAF6, ubiquitination of TRAF6, and TAK-1 phosphorylation occurred in the *lyn* ^−*/*−^ mast cells, which were not found in wild-type cells. However, stimulation of the Lyn-deficient mast cells with LPS did not increase the association of TRAF6 with TAK-1, and subsequent ubiquitination of TRAF6 and phosphorylation of TAK-1 were also inhibited. As a result of the impairment of the TAK-1 activation also phosphorylation of ERK1/2, p38 and JNK kinases and NFκB activation were inhibited in the Lyn-deficient mast cells [140]. This scenario resembled the impaired signaling of non-phosphorylated mutants of TLR4 which correlated with their low basal association with MyD88 that was not changed upon LPS treatment in contrast to the LPS-induced MyD88 recruitment by wild-type TLR4 [127]. It seems, therefore, that Lyn can modulate the TLR4/MyD88 and TRAF6/TAK-1 association/disassociation cycles required for optimal TLR4 signaling.

Lyn kinase has been found to associate with TRAF6 in LPS-stimulated mast cells [140]; however, the mechanism of this interaction has not been resolved. An indication on how the TRAF6 and Lyn kinase interaction could be regulated came from studies on TRAF6 functioning in LPS-stimulated human lung microvascular endothelial cells. In these cells, LPS binding to TLR4 triggers a cascade of tyrosine phosphorylations catalyzed by kinases of the Src family contributing to the disruption of the endothelial barrier integrity. In the endothelial cells, LPS stimulates the association of TRAF6 with Src and Fyn, but not with Lyn kinase [141], suggesting that identity of the Src family kinase engaged in TLR4 signaling can be cell type-dependent. The binding site for the Src kinases has been mapped to a proline-rich region of TRAF6. The assembly of the TRAF6-Src(Fyn) kinase complex requires the kinase enzymatic activity, while silencing of the *TRAF6* gene precludes activation of the Src kinases, which does not allow distinguishing which enzyme acts upstream of which [142]. In addition, during the association of TRAF6 with Src(Fyn), each protein can serve as a substrate for the catalytic activity of the other and their ubiquitination/phosphorylation is catalyzed [142]. If a similar relation holds for the TRAF6-Lyn interaction, Lyn would phosphorylate TRAF6 and stimulate the downstream TRAF6 signaling in LPS-treated cells while TRAF6 would catalyze Lys^63^-mediated ubiquitination of Lyn. Whether this polyubiquitination of Src family kinases affects their activity or association with TRAF6 remains unknown. It has been established that Lys^48^-linked ubiquitination and subsequent proteasomal degradation of Lyn controls its level in cells [143].

Recently, a newly discovered macrophage scaffolding protein Themis2 was found to bind Lyn kinase in LPS-stimulated RAW264 cells [144]. Analysis of the Themis2 role has provided evidence for the action of fine-tuning mechanisms of LPS-induced signaling. It was found that Themis2 underwent tyrosine phosphorylation on Tyr^660^ in LPS-stimulated cells and when phosphorylated could bind SH2 domain of Lyn kinase, however, the possibility of phosphorylation of Themis2 by Lyn has not been addressed. On the other hand, a proline-rich sequence of Themis2 binds constitutively the RhoGEF Vav. Therefore, clustering of Lyn and Vav at Themis2 could facilitate Vav phosphorylation by Lyn leading to stimulation of its GEF activity. The Themis2-Lyn-Vav axis has been found to stimulate selectively ERK and p38 activity but not that of JNK, NFκB, or IRF3. Eventually, the signaling events involving Themis2 and Lyn upregulate the production of TNF-α but not IL-6 or Cox2 in LPS-stimulated cells [144].

Some data indicate that not only plasma membrane-associated Lyn but also its intracellular fraction could be engaged in the signaling in LPS-stimulated cells. Lyn kinase was found to associate constitutively with MD-2 and to mediate its LPS-induced phosphorylation in the endoplasmic reticulum/endosomes of HEK293 expressing TLR4/MD-2 which suggested the MD-2 phosphorylation could be triggered after LPS internalization [145]. A key unresolved question regarding the proposed MD-2 phosphorylation by Lyn is how MD-2, apparently enclosed in the vesicle lumen, could be accessed by the kinase.

To summarize, stimulation of cells by LPS induces rapid activation of Lyn which can be involved in the initial steps of LPS-induced signaling by phosphorylating TLR4. The link between the Lyn and CD14 involvement in LPS-induced signaling, suggested by studies of Stefanova et al. [126] deserves further attention in view of the signaling properties of CD14 discussed in the previous chapter. Lyn kinase also associates with (and phosphorylates ?) TRAF6 and Themis2, downstream TLR4 signaling proteins, and can phosphorylate endosomal MD-2, probably only after LPS internalization. With the exception of the MD-2 phosphorylation, Lyn is likely to interact with other proteins and catalyze phosphorylation of its substrates at the plasma membrane. In all the cases mentioned above, Lyn activity stimulates the respective events of LPS-induced signaling pathways (Fig. 4, left). This, however, is not the only mode of Lyn participation in the signaling.

### Lyn kinase as a negative regulator of TLR4 signaling pathway

Lyn kinase is unique among the Src family kinases as it exerts both positive and negative regulatory action toward diverse signaling pathways [143, 146]. It has also been reported to act as a negative regulator of TLR4 signaling [147]. The studies were sparked by the finding that bone marrow-derived macrophages isolated from *lyn* ^−*/*−^ mice and stimulated with ReLPS produced more IL-6, TNF-α, and IFN-α/β than their wild-type counterparts. In agreement with those ex vivo studies, increased amounts of TNF-α, IL-6, and IFN-α/β were also found in the serum of *lyn* ^−*/*−^ mice injected with ReLPS. Those results indicated that Lyn is involved in the downregulation of both the MyD88- and TRIF-dependent pathways of TLR4. In search for the mechanism of the suppressory action of Lyn it was established that PI3-kinase and Akt kinase could be downstream targets of Lyn. It was inferred that the activity of PI3-kinase in LPS-stimulated cells led to phosphorylation of PI(4,5)P_2_ to PI(3,4,5)P_3_ in the plasma membrane and subsequent activation of Akt kinase. The latter was suggested to be responsible for the down-regulation of p38 phosphorylation eventually leading to decreased production of cytokines [147]. These data put PI3-kinase in a position of a negative regulator of TLR4 signaling (Fig. 4, right). Corroborating these results, wortmannin, a PI3-kinase inhibitor, augmented TNF-α and IL-6 production while a lack of SHIP1 phosphatase which dephosphorylates PI(3,4,5)P_3_ had an opposite effect. Recently, a contribution of PI3-kinase(s) to negative regulation of TLR4 pro-inflammatory signaling through the Akt-mTOR-Foxo1 signaling axis has been shown [148].

The association of Lyn with PI3-kinase(s) was described a decade earlier in LPS-stimulated human monocytes [149]. The enzymes co-immunoprecipitated and became activated in a coordinated manner within minutes of LPS action. Also, the level of PI(3,4,5)P_3_ increased markedly in LPS-treated cells, as found by analyzing P^32^-labeled lipid extracts by HPLC. Of note neutralizing CD14 with a specific antibody abrogated this LPS-induced PI(3,4,5)P_3_ production which indicated that CD14 and Lyn, two raft-enriched proteins, regulate PI3-kinase involvement and PI(3,4,5)P_3_ production in LPS-induced signaling.

There is a possible functional link between the CD14 involvement in LPS-induced signaling and PI(3,4,5)P_3_ production, as both control the endocytosis of TLR4. Expression of a kinase-dead p110δ isoform of the catalytic domain of PI3-kinase in mice inhibited LPS-induced endocytosis of TLR4 in bone marrow-derived dendritic cells [106]. The p110δ defect downregulated TRIF-dependent signaling and led to lower IFN-α, IP-10, and RANTES production with concomitant augmentation of MyD88/TIRAP-mediated signaling detected both in the mice and in the bone marrow-derived dendritic cells. The inhibition on TRIF-dependent signaling caused by the lack of the activity of p110δ isoform of PI3-kinase resembled that caused by the lack of CD14 in cells [28] but not the effects seen in Lyn-deficient macrophages where both MyD88- and TRIF-dependent signaling pathways of TLR4 were upregulated [147]. This indicates that proteins other than PI3-kinase p110δ co-operate with Lyn to regulate negatively both signaling pathways of TLR4.

Indeed, Lyn can exert its negative regulatory function by affecting many other proteins beside PI3-kinases. The list includes Cbp/PAG, a raft protein which is phosphorylated by Lyn and binds Csk kinase responsible for inactivation of Src family kinases [129]. Lyn also has a unique ability to phosphorylate the immunoreceptor tyrosine-base inhibitory motif (ITIM) of plasma membrane receptors such as FcγRIIb, paired immunoglobulin-like receptor B (PIR-B), signal regulatory protein-α (SIRPα), and CD22. These phosphorylated proteins recruit inhibitory phosphatases, such as protein phosphatase SHP-1/2 and SHIP1, a phosphatase of PI(3,4,5)P_3_ [150]. The latter enzyme does not seem to contribute to the Lyn-dependent negative regulation of TLR4 signaling [147] while other possibilities have not been examined in this context. However, the inhibitory role of FcγRIIb in TLR4-mediated responses was indicated [151].

In summary, the involvement of Lyn in LPS-induced signaling has both a positive role and generates a negative loop switching off the signaling (Fig. 4). Such a dual role of Lyn has been established previously for BCR and FcεRI signaling in mast cells. The positive versus negative action of Lyn in FcεRI-triggered signaling can depend on the intensity of stimulation of the receptor [152]. On the other hand, during activation of BCR, the two functions of Lyn need to be finely balanced and factors which determine the final outcome of Lyn activity are ill-defined, as both Lyn knockout mice and Lyn^up/up^ mice expressing constitutively active Lyn develop autoimmune diseases related to B-cell dysfunction [146]. Of special interest is that the positive role of Lyn detected in TLR4 signaling in mast cells contrasted with its negative role in TLR4 signaling in macrophages, since both series of experiments were performed on cells isolated form Lyn-deficient mice [140, 147]. Furthermore, in macrophages, a triple knockout of Hck, Fgr, and Lyn facilitated production of some cytokines [132]. These data suggest that the final outcome of the Lyn involvement in TLR4-triggered signaling could be cell type-specific and depend, e.g., on the receptors complexing with TLR4, like CD14 or CD36 (see below) or Fcγ receptors [29, 31, 153]. Taking into account that macrophages express CD14 at a high level while mast cells are CD14-deficient, it is tempting to speculate that if raft-residing CD14 is engaged in LPS-induced signaling, the negative regulatory function of Lyn rather than its indispensability for TLR4 signaling is emphasized. It is also plausible that Lyn regulates positively or negatively distinct components of TLR4 signaling pathways in a single cell type, resembling the Lyn involvement in BCR signaling [146]. Thus, Lyn could provide a subtle regulation of the amplitude and duration of the pro-inflammatory signaling triggered by LPS.

## Involvement of acid sphingomyelinase and other enzymes of sphingomyelin cycle in LPS-induced signaling

The accumulation of acid sphingomyelinase (ASMase) in the DRM fraction of RAW264 cells within 5–30 min of LPS treatment [84] points to the importance of sphingomyelin turnover in LPS-induced signaling. About half of the cellular pool of sphingomyelin is that in the plasma membrane, 80–90 % of which resides in the outer leaflet of the membrane and is enriched in rafts [154, 155]. Under the action of sphingomyelinases, sphingomyelin is hydrolyzed yielding ceramide, a multifaceted lipid which significantly changes the organization of the plasma membrane, affects the activity of several intracellular enzymes and is a precursor of other bioactive lipids, like ceramide-1-phosphate and sphingosine-1-phosphate [154, 156]. The generation of ceramide in the outer leaflet of the plasma membrane is linked with the activity of ASMase which translocates to the cell surface from intracellular compartments in response to diverse stimuli. Hydrolysis of sphingomyelin in the inner leaflet of the membrane is attributed to the activity of neutral sphingomyelinase (NSMase) [156–158]. ASMase and NSMase are activated rapidly in J774 and THP-1 cells under the action of LPS [83, 159, 160]. The production of ceramide by sphingomyelin hydrolysis in LPS-treated cells is also rapid, within minutes [160, 161], and is distinct from the TLR4-dependent de novo ceramide synthesis leading to prolonged ceramide accumulation observed in chronic diseases [162].

The data on the role of ASMase and ceramide in LPS-induced signaling are contradictory. Early studies indicated some similarities between LPS and exogenous ceramides, including long-chain ones, or ceramide generated in cells upon treatment with bacterial sphingomyelinase, in inducing the assembly of multimolecular complexes in the plasma membrane, activation of MAP kinases and production of selected cytokines in various cells, including RAW264 cells and murine macrophages [29, 161, 163, 164]. Furthermore, a positive role of endogenous ceramide generated by ASMase in the pro-inflammatory responses of cells to LPS was indicated. The ceramide was required for LPS-induced recruitment of TLR4 to rafts, activation of MAP kinases and TNF-α production in differentiated THP-1 cells, as all these events were inhibited by imipramine, a drug causing ASMase degradation, and were reversed by exogenous C2-ceramide [83]. In agreement, inhibition of ASMase by the SMA-7 drug prevented NFκB activation and release of pro-inflammatory mediators in THP-1 cells [159]. Recently, a positive influence of ASMase-generated ceramide on IL-6 production by RAW 264 cells after prolonged stimulation by low doses of LPS (1 ng/ml) and palmitic acid was found [165].

In contrast, studies performed on ASMase knockout mouse or murine macrophages and J774 cells after ASMase or NSMase silencing indicated that ceramide generated in LPS-stimulated cells downregulates the production of TNF-α and other cytokines [160, 166]. In accordance, infection of the ASMase-deficient mice with Gram-negative bacterium *Pseudomonas aeruginosa* resulted in a roughly tenfold higher release of pro-inflammatory cytokine IL-1β than in wild-type mice, and their death [167]. Exogenous C8-ceramide was found to inhibit production of pro-inflammatory cytokines, TNF-α, IL-6, and IL-12p40, by murine macrophages and pro-asthmatic IL-5, IL-10, and IL-13 by mast cells [168]. Accordingly, in a murine model of LPS- or *Staphylococcus aureus*-induced corneal inflammation, topical delivery of low doses of C6-ceramide in liposome formulation had a therapeutic effect supporting the anti-inflammatory properties of ceramide [169].

Ceramide generated in the outer leaflet of the plasma membrane can be converted back to sphingomyelin by sphingomyelin synthases 2 (SMS2) located in plasma membrane rafts/caveolae [156]. An impact of the SMS2 activity in the plasma membrane on pro-inflammatory signaling is yet to be proven unequivocally. Recent data indicate that in SMS2-deficient macrophages, enriched in ceramide and depleted in sphingomyelin, NFκB activation was attenuated and LPS-induced lung injury reduced [170, 171]. In SMS2-deficient mice, the serum level of IL-6 and TNF-α was lower compared to their wild-type counterparts [172]. These data underscore the anti-inflammatory outcome of changing the sphingomyelin/ceramide balance in the plasma membrane in favor of ceramide.

The molecular mechanisms of the action of the ASMase-generated ceramide in LPS-stimulated cells are not completely understood and their revealing is hindered by the fact that exogenous short-chain ceramides do not necessarily mimic the action of endogenous ceramides acylated with long-chain fatty acids which are produced by ASMase. It is known that the exofacial ceramide generated by ASMase dramatically alters the biophysical properties of the plasma membrane [154]. Ceramide molecules separate laterally into domains, thereby displacing cholesterol form rafts [173] and affecting the lateral distribution of plasma membrane proteins. For example, ceramide was found to facilitate clustering of FcγRII and CD95 in rafts [157, 174]. Following this scenario, one can assume that ceramide generated by ASMase in LPS-stimulated cells promotes mobilization of TLR4 to rafts and facilitates the CD14-TLR4 interaction leading to activation of pro-inflammatory signaling. Accordingly, binding of LPS to CD14 was found to be required for ceramide generation in THP-1 cells [83]. The ability of ceramide to induce redistribution of plasma membrane proteins most likely accounts for the observed recruitment of selected cell surface proteins toward CD14 creating conditions for activation of selected signaling pathways in cells exposed to exogenous ceramides [29, 163].

The above scenario is in contrast with reports indicating a negative influence of ASMase-generated ceramide on the production of pro-inflammatory mediators, like TNF-α [160, 166]. The ceramide inhibited maturation of TNF-α at the stage of the cleavage of TNF-α precursor to its active 17-kDa soluble form by the TNF-α converting enzyme (TACE) [166], which also associated with rafts [175]. The ceramide negatively regulated TACE activity, which was tentatively linked with changes of TACE partitioning to rafts in ASMase-deficient cells. In addition, ASMase-generated ceramide could also affect cellular trafficking of the TNF-α precursor [166]. The mechanisms of the negative influence of ceramide on the production of other cytokines in LPS-stimulated cells are unknown, albeit inhibition of the LPS-induced activity of NFκB and AP-1 by exogenous C-2 ceramide has been reported in RAW264 cells [176]. Possibly, the impact of the ASMase-generated ceramide on the associations of TLR4 with rafts also needs further studies. The shift of the balanced from sphingomyelin toward ceramide in the plasma membrane seems to negatively affect the association of TNF-α receptor with rafts [170]. Bearing in mind the positive effect of ceramide on the association of FcγRII and CD95 with rafts, the lipid seems to be able to both facilitate and hinder the partition of various receptors to rafts. In accordance, recent studies on transferrin receptor ligated with transferrin indicated that ASMase-generated ceramide inhibited receptor partitioning to rafts and facilitated its endocytosis by clathrin-coated pits [177]. The latter is in agreement with the ability of exofacial ceramide to induce inward curvature of the plasma membrane and subsequent budding of endocytic vesicles [178]. Taking into account, the dynamic association of TLR4 with rafts followed by its endocytosis in LPS-stimulated cells, one can expect a complex impact of sphingomyelin/ceramide turnover on the receptor translocations in the plasma membrane which could change in the course of TLR4 activation. Such complexity could account for the discrepant effects of the interference with the turnover of sphingomyelin in LPS-stimulated cells.

## Proteasome as newly identified raft component involved in LPS signaling

A recent proteomic analysis has revealed that following LPS action, numerous proteins involved in protein ubiquitination and several proteasome subunits are recruited to the DRM fraction of RAW264 cells [84]. Furthermore, p105 protein was identified as a proteasomal substrate in the DRM fraction of LPS-stimulated cells. Degradation of this protein releases Tpl2 kinase which phosphorylates MEK and leads to ERK activation, and both phosphorylated MEK and ERK kinases were found to be confined to the DRM fraction of RAW264 cells [84, 117]. Thus, these studies suggest that the plasma membrane rafts are sites of the activity of proteasomes linked to ERK activation in LPS-stimulated cells. It has also been shown that LPS alters the composition of proteasomes, and that proteasomal degradation of several signaling proteins regulates both MyD88- and TRIF-dependent generation of pro-inflammatory mediators [179], with the proteolysis of IκB by 26 S proteasome being the best characterized. Remarkably, 26 S proteasome is activated by LPS in the DRM fraction of RAW264 cells [84]. Proteasome activity is a novel function linked to rafts and further studies are required to reveal how proteasomes associate with the plasma membrane and are activated in LPS-stimulated cells.

## Involvement of CD44 and Hsp70 in TLR4 signaling triggered by LPS and non-microbial ligands

CD44, the hyaluronan receptor, is another raft protein likely to co-operate with TLR4. This co-operation regulates the pro-inflammatory responses induced by LPS and also those observed in the absence of microbial stimuli. During trauma fragments of hyaluronan are released from the extracellular matrix and, after binding to CD44, induce expression of pro-inflammatory genes in a TLR4/MD-2-dependent manner. Only some of these genes are those activated by LPS as well, as shown in vivo by studying skin samples of sterile-injured mice [180]. In those studies, CD44 was identified as an important intermediate in the hyaluronan-induced TLR4 activation. However, in the presence of microbial PAMPs, CD44 negatively regulates cytokine production: macrophages derived form *Cd44* ^−*/*−^ mice and exposed to LPS or ligands for TLR2, TLR3, TLR6, TLR8, or TLR9 produced more cytokines compared with *Cd44* ^+*/*+^ cells [181]. In agreement with those molecular data, CD44-deficient mice were more susceptible to LPS-induced shock [182, 183]. The protective role of CD44 against excessive inflammatory responses to LPS was ascribed to expression of negative regulators of TLR4 signaling, IRAK-M, Toll-interacting protein, and AD20 [182, 183]. Hyaluronan also acts through TLR4 to inhibit TLR3-dependent inflammation [184].

Hyaluronan is one of alarmins or DAMPs (damage-associated molecular patterns), endogenous molecules released from injured or dead cells and activating pattern recognition receptors for production of pro-inflammatory mediators in a similar manner as PAMPs do. This class of molecules also includes heat shock proteins [185]. After exposure of cells to a stress even without LPS stimulation, cytoplasmic Hsp70 incorporates into the plasma membrane in the raft region judging from its partition to the DRM fraction. The protein spans the membrane exposing only a small portion of its C-terminus to the cell exterior and is released in the membrane-bound form by exocytosis or membrane shedding. When reintroduced into J774 cell culture such membrane-associated Hsp70 induces production of TNF-α [186]. Soluble Hsp70 can also associate with/incorporate in the plasma membrane of macrophages with a preference toward the DRM fraction and stimulate phagocytosis [187], but its ability for TNF-α induction is much weaker than that of the membrane-bound form [186]. The Hsp70-bearing membranes used in those studies were prepared from eukaryotic HepG2 cells; therefore, the pro-inflammatory activity observed can safely be ascribed to Hsp70 itself rather than to contaminating microbial components, as suggested earlier [188]. Notably, the relation between Hsp70 and TLR4 activity was not addressed in the above studies. However, preincubation of THP-1 cells with Hsp70 reduced the NFκB activation by subsequent LPS treatment, an effect known as endotoxin tolerance, which indicates a cross-talk between the signaling cascades triggered by the DAMP and PAMP [189].

The participation of Hsp70 in LPS-triggered pro-inflammatory responses is supported by a diversified line of data. In LPS-stimulated cells, Hsp70 and Hsp90 were among the few proteins found in close proximity to GM1 and TLR4 in the plasma membrane by FRET analysis, and accumulated together with TLR4 in the DRM fraction [33, 69, 190]. LPS and the febrile-range temperature that accompanies first days of sepsis induced Hsp70 expression and its release outside RAW264 cells. Concomitant exposure of mice to febrile-range hyperthermia and intratracheal administration of LPS also caused an elevation of Hsp70 in a cell-free lung lavage greater than hyperthermia or LPS alone [191]. It has been also demonstrated that Hsp70 and Hsp90 bind LPS [33]. The interaction of Hsp70 and Hsp90 with LPS, and possibly also with lipopeptides, is probably mediated by the hydrophobic interactions that otherwise facilitate the interactions of the Hsp with their substrate proteins. Thus, it seems likely that Hsp70 acts as an LPS/lipoprotein acceptor donating them to TLR4 or TLR2 and inducing production of pro-inflammatory mediators after incorporation into plasma membrane rafts. It has been also hypothesized that rafts can serve as platform which govern phagocytosis triggered by Hsp70 [187], leaving an open question of a possible contribution of this process to the pro-inflammatory activity of Hsp70.

## CD36, a newly discovered player in TLR4 signaling triggered by non-microbial ligands

CD36, a class B scavenger receptor, is a plasma membrane protein of an unusual hairpin-like structure with both its N- and C-termini directed toward the cytoplasm and connected by a large extracellular ligand-binding loop. The both cytoplasmic tails of CD36 are palmitoylated, which modification facilitates the association of CD36 with rafts isolated as the DRM fraction [55, 56]. Those early studies indicated also that CD36 associates with Lyn kinase in rafts, and this interaction has recently been proven to be pivotal for controlling TLR signaling triggered by ligands of endogenous origin [153]. After recognition of oxidized low-density lipoprotein (oxLDL) or β-amyloid, CD36 undergoes phosphorylation on C-terminal Tyr^436^ and associates with Lyn, thereby inducing formation of a heterotrimeric complex of CD36, TLR4, and TLR6. Once clustered, TLR4/TLR6 trigger signaling pathways which engage MyD88 and TRIF, activate inflammasome and induce production of an array of pro-inflammatory mediators. Signaling from the CD36/TLR4/TLR6 complex requires dynamin-mediated endocytosis. This is in agreement with studies showing that CD36-mediated endocytosis provides the major route for the oxLDL uptake by human and mouse macrophages [120, 192]. The molecular mechanisms of CD36-induced internalization are divergent but the prevailing one has been characterized by Heit et al. [120], and discussed above as a possible model of CD14-mediated uptake of TLR4/LPS. The apparent link between CD36 activity and endocytosis which controls signaling of the TLR4/TLR6 complex recalls the participation of CD14 in endocytosis of TLR4 which governs TRIF-dependent signaling in LPS-stimulated cells. The mechanism of the cross-talk among CD36, Lyn, TLR4, and TLR6 remains unknown; however, the involvement of CD36 and Lyn indicates a raft-based regulation of these events [153]. Of note, that newly detected link between CD36 and TLR4/TLR6 activity was described in microglia and monocytes/macrophages activated not by microbial PAMPs but by endogenous ligands [153, 193] whose accumulation is a hallmark of atherosclerosis and Alzheimer’s disease.

CD36 also participates in the recognition of microbial PAMPs. It is a multipurpose protein which binds and mediates internalization of Gram-positive and Gram-negative bacteria as well as LPS [35, 194, 195]. Human CD36 can activate JNK1/2 kinase and IL-8 production in cells exposed to LPS or *E. coli* independently of TLR4 [195]. Therefore, the action of LPS as a possible contaminant in oxLDL samples needs to be considered when studying the pro-inflammatory activity of oxLDL, as indicated recently by Kannan et al. [196]. A line of data indicates that the protein functions also as a co-receptor which delivers other bacterial components to TLRs. CD36 serves as an acceptor of lipoteichoic acid and diacylated peptides and subsequently associates with TLR2/TLR6 complex, likely using rafts as regulators of the CD36–TLR2–TLR6 proximity [197]. A complex of CD36, TLR2/TLR1, and CD11/CD18 likely formed in rafts has been shown to mediate pro-inflammatory responses to the tetra- or penta-acylated LPS chemotypes which do not activate TLR4 [198]. The pro-inflammatory reaction to these ligands can be further upregulated by the presence of oxLDL [199]. Taken together, the data indicate that CD36 binds distinct “self-” and “non-self” ligands and contributes to activation of diverse TLRs and other co-receptors. Both the raft localization and the endocytic activity of CD36 seem crucial for its involvement in TLR signaling pointing to similarities between functioning of CD36 and CD14.

## Concluding remarks

Ample data point to the importance of raft integrity for pro-inflammatory TLR4 signaling. These include the disturbances of the signaling caused by manipulation of the cholesterol level in the plasma membrane or by interference in the sphingomyelin/ceramide balance. Furthermore, several cell surface proteins accumulated in plasma membrane rafts, like CD14, CD44, and CD36 participate in the recognition of LPS and non-microbial components which subsequently activate TLR4. Others, like Lyn kinase anchored in the inner leaflet of the rafts, are involved in TLR4-triggered signaling cascades. Surprisingly, the importance of the raft localization of these proteins for their involvement in TLR4 signaling has only rarely been addressed. A common feature of the CD14 and CD36 engagement in TLR4-induced signaling is their contribution to the internalization of activated TLR4 and TLR4/TLR6. This suggests that the endocytosis of the receptors could be a major phenomenon governed by raft-associated proteins during stimulation of cells with LPS or non-microbial components activating TLR4. Rafts can serve as platforms clustering together a group of proteins, like ITAM-containing proteins, Lyn and Syk kinases, and PLCγ2 controlling the uptake of TLR4 to trigger the TRIF-dependent signaling pathway.
